# What do parents perceive are the barriers and facilitators to accessing psychological treatment for mental health problems in children and adolescents? A systematic review of qualitative and quantitative studies

**DOI:** 10.1007/s00787-016-0930-6

**Published:** 2017-01-04

**Authors:** Tessa Reardon, Kate Harvey, Magdalena Baranowska, Doireann O’Brien, Lydia Smith, Cathy Creswell

**Affiliations:** School of Psychology and Clinical Language Sciences, University of Reading, Reading, UK

**Keywords:** Mental health, Children, Adolescents, Treatment access, Barriers

## Abstract

A minority of children and adolescents with mental health problems access treatment. The reasons for poor rates of treatment access are not well understood. As parents are a key gatekeeper to treatment access, it is important to establish parents’ views of barriers/facilitators to accessing treatment. The aims of this study are to synthesise findings from qualitative and quantitative studies that report parents’ perceptions of barriers/facilitators to accessing treatment for mental health problems in children/adolescents. A systematic review and narrative synthesis were conducted. Forty-four studies were included in the review and were assessed in detail. Parental perceived barriers/facilitators relating to (1) systemic/structural issues; (2) views and attitudes towards services and treatment; (3) knowledge and understanding of mental health problems and the help-seeking process; and (4) family circumstances were identified. Findings highlight avenues for improving access to child mental health services, including increased provision that is free to service users and flexible to their needs, with opportunities to develop trusting, supportive relationships with professionals. Furthermore, interventions are required to improve parents’ identification of mental health problems, reduce stigma for parents, and increase awareness of how to access services.

**Electronic supplementary material:**

The online version of this article (doi:10.1007/s00787-016-0930-6) contains supplementary material, which is available to authorized users.

## Introduction

Mental health disorders are common among children and adolescents, with an estimated prevalence rate of 13.4% [1]. Youth is a time of heightened risk for mental health disorders, with half of all lifetime mental health disorders emerging before the age of 14 years [2]. Moreover, the negative impact of poor mental health early in life extends into adulthood, predicting poor academic outcomes [3], increasing the risk of subsequent mental health problems [4] and high rates of mental health service use [5], reducing life satisfaction [6], and creating a heavy economic burden for society [7].

In recent decades, there has been a rapid growth in the development of evidence-based treatments for mental health disorders in childhood and adolescence; and the lasting benefits of intervening early are well established [8, 9]. However, poor rates of treatment access have been repeatedly reported, and national surveys in the UK, Australia, and USA have estimated that only 25–56% of children and adolescents with mental health disorders access specialist mental health services [10–12], with particularly low rates of access for internalising compared with externalising problems [10, 12].

In an effort to explain the unmet need in relation to childhood mental health disorders, studies have often focused on identifying predictors of service use. Family and child characteristics, including ethnicity, family socioeconomic, and insurance status, living in an urban or rural area, and severity of the child’s problems have all been implicated in determining the likelihood of service utilization. Overall studies suggest that being Caucasian [13, 14], having insurance coverage (in the USA) [15, 16], living in an urban area [17], and having a child with more severe mental health problems [12] increases the likelihood of a family accessing treatment. While these studies shed some light on who accesses treatment, they tell us little about the reasons for discrepancies in service use or the processes underlying accessing treatment.

An alternative approach draws on models of help-seeking behaviour to conceptualise different stages and processes involved in accessing treatment for mental health problems in children and adolescents [16, 18, 19]. Specifically, factors have been explored that underlie the distinct stages of (1) parental recognition of difficulties, (2) the decision or intention to seek help, and (3) contact with services. Studies of parental recognition suggest that parents who perceive that a problem exists and think that the problem has a negative impact on family life are more likely to seek help and access mental health services for their children than those who do not recognise a problem or its negative impact [20, 21]. Parental attitudes surrounding mental health and mental health services have been shown to influence help-seeking decisions—in particular, beliefs that mental health problems are caused by child’s personality or relational issues [22], negative perceptions of mental health services [18], and perceived stigma associated with mental health problems [23, 24] have all been associated with reduced help-seeking behaviour. Similarly, ‘logistical’ factors (such as transport access and flexibility of appointment system) have been shown to influence the likelihood of a family having contact with services [25, 26]; and a parent sharing concerns about a child’s mental health with a primary care practitioner has also been shown to improve access to mental health services [27, 28].

Together these studies highlight the key ‘gatekeeper’ or ‘gateway provider’ [29], role parents can play in treatment access for mental health problems for children and adolescents, and point towards numerous potential barriers parents may face in the process of seeking and obtaining help. However, to improve access to treatment, it is important to establish parents’ own views on the factors that may help and hinder access. Indeed, studies focusing on ongoing treatment engagement (i.e., continuing treatment after initial access) have identified key factors that parents perceive to be barriers to treatment attendance [30, 31], and thereby highlight areas to target to improve treatment retention. Therefore, similarly, identifying what parents perceive to be the barriers and facilitators to the initial access to treatment would highlight areas to target to improve rates of access.

A recent systematic review synthesised findings across studies that reported young people’s perceptions of barriers and facilitators to accessing mental health treatment [32]. However, given that children and adolescents are rarely able to seek and access help alone, it is equally important to establish parents’ corresponding views; a review of parents’ perceptions of barriers and facilitators to treatment access has not been conducted to date. The purpose of this study is to systematically review studies that report parents’ perceptions of barriers and/or facilitators to accessing treatment for mental health problems in children and adolescents. The review synthesises findings across both quantitative and qualitative studies, incorporating studies that focus on specific mental health disorders, as well as those considering emotional and/or behavioural problems more broadly. The review focuses on access to psychological treatments (rather than medication), and is concerned with the processes of both seeking and obtaining help through specialist mental health services, as well as primary care and school settings.

## Method

A systematic literature review was conducted following PRISMA guidelines [33].

### Literature search

Four electronic databases were searched in October 2014. The NHS Evidence Healthcare Database was used to run a combined search of Medline, PsychInfo, and Embase; and the Web of Science Core Collection was searched separately. With reference to relevant literature and previous reviews, search terms to reflect the following four key concepts were generated: barriers/facilitators; help-seeking; mental health; and parents/children/adolescents. Search terms within each of these four categories were combined using ‘AND’ to search titles/abstracts. Searches were limited to articles published in English (see Electronic supplementary material 1 for details of search strategy).

Additional hand searching methods were also employed. The reference lists of relevant articles in the field identified through the database search were scanned for additional studies. Citations of relevant articles were then searched to help identify more recent studies not yet included in the electronic databases.

### Study eligibility

Inclusion and exclusion criteria were drafted and then refined after piloting using a small sample of papers (see Electronic supplementary material 2 for details of full criteria). A study was selected for inclusion if:

1. parents/caregivers of children/adolescents were participants. Studies were excluded if the mean age of the children/adolescents was over 18 years or the sample included adults over 21 years;
2. parents’/caregivers’ perceived barriers/facilitators to accessing treatment for mental health problems in children or adolescents were reported. Studies that only reported barriers/facilitators perceived by children/adolescents were not included;
3. the study was published in English in a peer-reviewed journal. Reviews were excluded.

Studies reporting quantitative or qualitative data (or both) were included. There was no requirement relating to the nature of the mental health problem; studies focusing on either a specific mental health disorder (e.g., depression, ADHD) or behaviour and/or emotional problems more generally were included. However, studies that only reported factors associated with or predictors of help-seeking or service use were not included. Similarly, studies reporting outcomes of an intervention targeted at overcoming one or more barriers to help-seeking were not included. As the focus of the review was barriers and facilitators to accessing psychological treatments within the general population, studies focusing on access within a special population (e.g., children/adolescents with intellectual disability and children/adolescents with mental health problems in the context of a specific physical health condition); and studies specifically addressing access to medication or inpatient psychiatric care (as these would rarely be the first-line treatments), or parenting programmes not specifically targeting mental health problems in children/adolescents were not included.

### Study selection

**Fig. 1:**
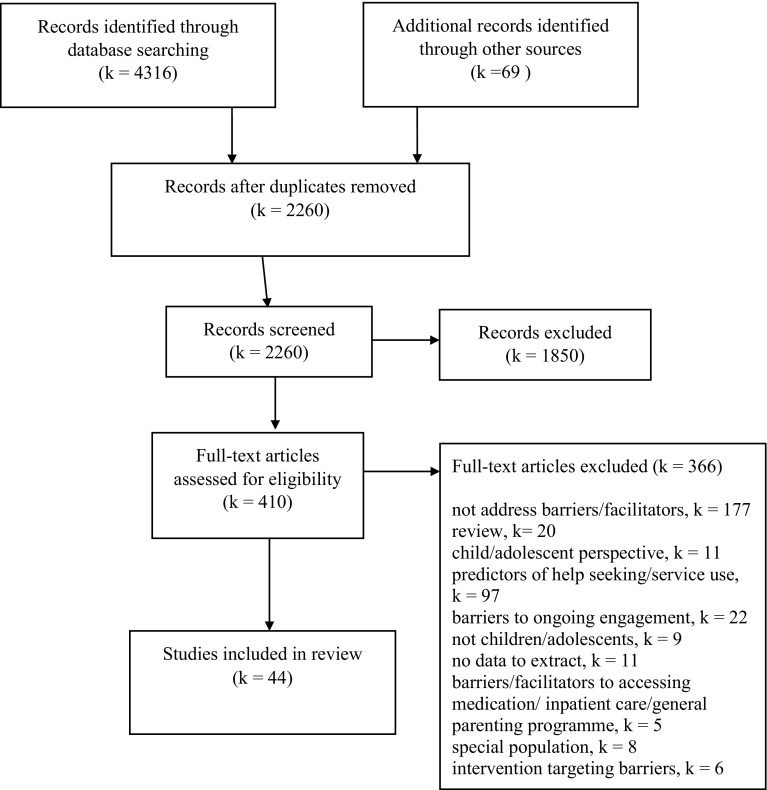
PRISMA flowchart

Details of the study selection process are provided in the flowchart in Fig. 1. The combined electronic database search retrieved 4316 records, leaving 2191 records after duplicates were removed. Hand searching identified additional 69 potentially eligible papers. Two independent reviewers (TR and MB/LS) then screened the 2260 titles and abstracts and excluded studies using the criteria detailed above. Agreement between reviewers was good (85% agreement). If either reviewer selected the study for potential inclusion, the full paper was sourced. Two reviewers (TR and MB/LS) then independently assessed the 410 full papers for inclusion, and if the study failed to meet inclusion criteria, the primary reason for exclusion was recorded. In cases of disagreement in inclusion/exclusion judgement, the paper was passed to a third independent reviewer (CC) and a final decision was agreed. In total, 44 studies met criteria for inclusion in the review.

### Data extraction

Two standard data extraction forms were developed: one for studies reporting quantitative data and a second for studies reporting qualitative data. The extraction forms were drafted and then refined after the initial piloting by reviewers. Two reviewers (TR and MB or LS) then independently extracted data for each included study, using the corresponding extraction form (or in the case of mixed method studies, using both forms). Any discrepancies in extraction were discussed between the two reviewers, and if there were differences in interpretation, a third reviewer (CC) was consulted and a consensus agreed.

The following information was extracted for each included study: (1) methodology used (quantitative, qualitative, or mixed methods); (2) country of study; (3) study setting (e.g., mental health clinic, school); (4) parent/caregiver characteristics (number and percentage of mothers); (5) child/adolescent characteristics (age range, mental health status, mental health service use, type of mental health problem, or disorder); and (6) whether the study targeted a particular ethnic group or urban/rural population. For studies that collected and analysed quantitative data, details relating to the measure of barriers/facilitators were also extracted (e.g., name of measure, number and format of items, content of items [e.g., subscales, broad areas covered], whether it is a published measure or developed for the study). Where studies reported qualitative data, the method used to collect data (e.g., focus groups, interviews) and areas of relevant questioning were recorded. Finally, information relating to parental perceived barriers/facilitators was extracted from the results section, including the name of each reported barrier and facilitator and associated evidence (e.g., number of participants who endorsed the barrier/facilitator, participant quotes).

### Quality rating

The quality of included studies was assessed using modified versions of the two checklists developed by Kmet and colleagues [34]. One checklist was specifically designed for use with quantitative studies and the other for use with qualitative studies, thus allowing corresponding evaluations of different study designs; and studies that used mixed methods were assessed using both checklists. Items on the checklist for assessing the quality of quantitative studies that were not relevant to studies included in this review were removed (e.g., ‘If interventional and random allocation was possible, was it described?’); and the wording of other items was tailored for the purpose of this review (e.g., ‘Measure of barriers/facilitators well defined’). Items on the checklist for assessing the quality of qualitative studies were slightly modified to incorporate Dixon-Woods’ [35] prompts for appraising qualitative research (e.g., ‘Are the research questions suited to qualitative inquiry?’). Items on both checklists are rated on a three-point scale (yes = 2, partial = 1, and no = 0), with a maximum score of 20 on the quantitative checklist and 18 on the qualitative checklist. Items on each checklist that related to methods of data collection, data analyses, and conclusions drawn were judged specifically in relation to the part of the study that focused on parental perceived barriers/facilitators (see Electronic supplementary material 3 for modified versions of checklists). Based on the final score, studies were classified into three groups to reflect the overall spread of quality ratings across studies, including: low (quantitative: 0–12; qualitative: 0–11), medium (quantitative: 13–16; qualitative: 12–15), and high (quantitative: 17–20; qualitative: 16–18) quality.

Two reviewers (TR and MB/LS/KH) independently assessed the quality of each included study. Twenty studies were rated using the checklist for quantitative studies, twenty-two studies were assessed using the checklists for qualitative studies, and two studies that used both qualitative and quantitative methods were assessed using both checklists. The two reviewers discussed any discrepancies in ratings, and, if necessary, consulted a third reviewer (KH or CC) to reach a final decision.

### Data synthesis

A narrative synthesis was conducted, drawing on the framework and techniques described in ‘ERSC Guidance on Conducting Narrative Synthesis’ [36]. Initially, preliminary syntheses of the quantitative data and the qualitative data were each conducted separately. Tabulated quantitative data were reorganised to group findings according to reported perceived barriers/facilitators, and then, a code was attached to each individual reported barrier/facilitator. Data were reorganised according to the initial codes, and then, an iterative process was adopted in which codes were refined, and grouped into overarching emerging barrier/facilitator themes. Tabulated qualitative data were then coded and organised into barrier/facilitator themes, following the same iterative process. The next step was to develop a ‘common rubric’ [36] to amalgamate quantitative and qualitative findings. This involved refining quantitative and qualitative codes, to develop a single-coding framework, that described and organised the barriers/facilitators identified across all studies.

To facilitate the process of comparing and contrasting findings across studies, and in particular to examine variation in the number of participants who endorsed particular barriers/facilitators, further ‘transformation’ [36] of quantitative data was performed. First, where applicable, responses on Likert response scales were converted into ‘percentage endorsed’ by summing positive responses (e.g., summing number of ‘agree’ and ‘strongly agree’ responses). Next, the ‘percentage endorsed’ for each barrier/facilitator was examined and categorised into three groups according to the relative overall spread of endorsement rates across studies [‘small’ (0–10%), ‘medium’ (10–30%), and ‘large’ (more than 30%)]. Graphical representations were then used to display the percentage of studies that reported individual barriers and facilitators, illustrating the percentage of quantitative studies in which the barrier/facilitator was reported by at least a ‘medium’ percentage of participants, as well as the percentage of qualitative studies that reported corresponding barriers/facilitators. Similarities and differences in study findings, and the relationship between individual barriers/facilitators, and barrier/facilitator themes, were further explored using data extracted in relation to study characteristics (e.g., study setting, sample characteristics, and mental health service use).

Finally, to assess the robustness of the data synthesis, a sensitivity analysis was performed in which findings from studies assessed to be of ‘low’ quality were removed, and the remaining data were re-examined to determine if the codes, key themes, and conclusions remained unchanged.

One reviewer (TR) conducted the data synthesis, with regular discussion with team members (CC, KH, and DO’B) to agree interpretation of data and formulation of codes and themes.

## Results

### Description of included studies

In total, 44 studies were included in the review, with 20 studies providing quantitative data, 22 providing qualitative data, and two providing both quantitative and qualitative data. Details related to the study characteristics are provided in Tables 1 and 2.

**Table 1:** Study characteristics of quantitative studies

Study	N	Age range	Country	% mothers ethnicity urban/rural	Study setting	Mental health status	Mental health service use	Measure of perceived barriers/facilitators	Quality rating (0–20)
Berger-Jenkins (2012) [64]	70	5–17	USA	Hispanic (57.1%) African American (34.3%)	primary care and mental health clinic waiting room	ADHD diagnosis (self reported/chart review)	No current treatment	5 items yes/no response; system-related barriers (responses to other items not included)	18 (high)
Bussing (2003) [47]	91	5–11	USA	African American (50%)	schools	ADHD diagnosis (screening; diagnostic assessment)	No treatment (previous 12 months)	20 items yes/no response; system-related barriers; stigma; perceived need; financial; negative expectations	19 (high)
Cheng (2013) [39]	67	5–17	UK	Chinese (100%)	Chinese language schools	7% borderline/abnormal range (SDQ)		1 item; reasons not seek professional help	12 (low)
Dempster (2013) [23]	102	5–8	USA	Mothers (95%) Rural	Primary care (appointment check in)	8.8% above clinical cut-off (PSC)		Obstacles to Engagement Scale 14 items four-point response scale; barriers to attending parenting class for child behaviour problems personal/family stressors; trust/relevance intervention; intervention demands; time/scheduling demands	19 (high)
Eapen (2004) [40]	205 (out of 325)	United Arab Emirates		Community			Not sought help	1 item; reasons not seek specialist help	8 (low)
Girio-Herrera (2013) [50]	306	5	USA	Caucasian (94.4%) Rural	Elementary schools	‘At risk’ (above cut-off on BASC-2)	11% receiving professional help	Barriers to Participation Scale (modified) 44 items five-point response scale; relevance; demands/issues; relationship; stress/obstacles	18 (high)
Gould (2009) [76]	17	13–19	USA		High school	‘At risk’ (above cut-off SIQ, or endorse relevant SIQ and BDI items)	Not follow through with referral and seek treatment	Help-seeking utilization questionnaire 18 items; reasons for non-use of services: shame; self-efficacy and structure	18 (high)
Harrison (2004) [72]	66		USA	African American (79%) Hispanic (12%) Urban	Referred to mental health clinic	Clinically significant mental health need (CPRS)	Not attend first appointment at mental health clinic	CASA; reasons for non-attendance at mental health clinic: concrete obstacles; receptivity to services; attitudes towards professional helpers; previous experience with services	15 (medium)
Harwood (2009) [37]	110	3–6	USA	Caucasian (63%) African American (20%)	Paediatric primary care waiting rooms	34% above cut-off on ECBI (disruptive behaviour)	24% never received mental health service 62% only read information	Survey of parental attitudes (some relevant items) five-point response scale; barriers and facilitators to obtaining recommended services	16 (medium)
Hickson (1983) [70]	149		USA	Mothers (100%)	Paediatric primary care waiting room	Report psycho-social concern	Not communicated psycho-social concern with paediatrician	Part of interview reasons for not communicating psycho-social concerns with paediatrician	8 (low)
Ho and Chung (1996) [77]	74	0–15	Hong Kong		Consecutive referrals to psychiatric clinic	Range of psychiatric diagnoses	Referred to psychiatric clinic; no prior access	Part of interview difficulties encountered in help-seeking process	12 (low)
Larson (2013) [38]	55	2–17	USA	African American (98%)	Referrals within a paediatric clinic to a mental health clinic	Range of mental health problems	64% followed-up with referral; 36% not attend initial evaluation/not schedule first appointment	23 items; perceptions of mental health treatment and potential barriers to seeking mental health care	18 (high)
McKay (2002) [62]	159		USA	African American (81%) Latino (11%) Urban	Consecutive referrals to mental health clinic	Disruptive behaviour problems	Referred to mental health clinic; 45% prior treatment	Adapted CASA barriers to services use	11 (low)
Meredith (2009) [49]	329	13–17	USA	Hispanic (45.8%) Black (33.9%) White (16.7%)	Primary care waiting room	Depression diagnosis (*n* = 162) [49] matched sample with no depression diagnosis (*n* = 167) [49] (diagnostic interview)		Part of interview 6 items five point response scale; imagine need or want care 6 months in future, rate following barriers: cost; what others think; difficulty making appointment; personal responsibilities; no good care available; not want to	19 (high)
Mukolo (2011) [51]	175	4–17	USA	African American (100%) urban (33%)	Medicaid enrolment data (subsample)	Mean score above clinical cut-off (CBCL-internalising and externalising scales)	Mental health service use previous 6 months	14 items yes/no response; rate if items made it difficult to obtain services or prevented their child getting services in previous 6 months location/time; provider/payer; family perception barriers	19 (high)
Murry (2011) [46]	163	13+	USA	Mothers (100%) African American (100%) Rural	Longitudinal study (wave 8) random sample of African American families from school lists	23% clinical/borderline range (CBCL)	10% reported seeing someone for child’s emotional/behaviour problem	Mothers’ Perceptions About Help Seeking three-point response scale; mother’s/child’s lack of willingness; cultural/general mistrust service providers; lack of social support; stigma	17 (high)
Owens (2002) [26]	116		USA	African American (81.9%)	School-based early intervention study (grade 1) follow-up across control and intervention groups (grade 7)	Reported need for mental health service 71.3% no diagnosis; 16.5% conduct disorder; 12.2% other diagnosis (DISC-IV)	35.6% accessed mental health services (one year previously)	15 items; reasons not accessed care: structural; perceptions about mental health problems; perceptions about mental health service	19 (high)
Pavuluri (1996) [53]	34	30–60 months	New Zealand		Preschools subsample who reported barrier/s to help-seeking		Reported need for mental health service	16 items; reasons for not seeking help when help was needed	15 (medium)
Sawyer (2004) [52]	286	4–17	Australia		National survey diagnostic interview	ADHD diagnosis (diagnostic interview)	Reported not attending services in previous 6 months	Part of service use survey reason for not attending services	11 (low)
Sayal (2015) [41]	162	10	UK		Part school-based intervention trial	High risk for ADHD (screening at age 5)	Specialist service use in previous 5 years (*n* = 81) [41] matched sample with no specialist service use in previous 5 years (*n* = 81) [41]	Part of Children Services Interview aspects of service provision perceived as presenting a barrier to service use (educational and health) availability of information about where to seek help; attitudes and communication of professionals; practical issues (e.g., cost, getting to appointments, confidentiality); what other people would think	17 (high)
Shivram (2009) [59]	420	5–16	UK		National survey	Conduct disorder diagnosis (DAWBA)	25.2% specialist child mental health service use	15 items; reasons that prevented service access (previous 12 months) when concerned	17 (high)
Thurston (2008) [48]	194	2–17	USA	African American (51.5%) Caucasian (48.5%)	Community (responders to flyers and adverts)		19.4% past service use	Barriers to Treatment Utilization Likert response scale structural barriers (accessibility, availability); attitudinal barriers (acceptability, accountability)	14 (medium)

*n* number of parents/caregivers, *age range* age range of children/adolescents, *SDQ* Strengths and Difficulties Questionnaire, *PSC* Pediatric Symptom Checklist, *BASC*-*2* Behaviour Assessment System for Children Second Edition, *SIQ* Suicidal Ideation Questionnaire, *BDI* Beck Depression Inventory, *CPRS* Conners Parent Rating Scale, *ECPI* ECPI Intensity Scale, *CASA* Child and Adolescent Services Assessment; *CBCL* Child Behaviour Checklist, *DISC-IV* diagnostic Interview Schedule for Children Version 4, *DAWBA* Development and Well-Being Assessment

**Table 2:** Study characteristics of qualitative studies

Study	N	Age range	Country	% mothers ethnicity rural/urban	Study setting	Mental health status	Mental health service use	Method of data collection (relating to perceived barriers/facilitators)	Quality rating score (0–18)
Boulter and Rickwood (2013) [44]	15	<18	Australia	Mothers (93%)	Local mental health services, parent support groups, community mental health education group		Previous help seeking for mental health problems	Semi structured interview; address type of help sought, help-seeking process and decision to seek help	18 (high)
Boydell (2006) [60]	30	3–17	Canada	Mothers (80%) Rural	Community meetings; two rural areas	Previous emotional/behavioural disorder diagnosis		Indepth interview; address issues related to access to mental health care, including barriers and facilitators	14 (medium)
Bradby (2007) [63]	(a) 35 (b) 7 (c) 6	a. b. 6–14 c. 5–13	UK	South Asian (100%) Urban	(a) Community sample, various sources (b) Current/recent specialist mental health service users (c) professionals (GPs, health visitors, schools) identified families where child would benefit from specialist mental health service	(a) (b, c) range of mental health problems	(a) (b) Current/recent mental health specialist service users (c) ‘Potential’ service users (not yet received referral)	(a) Community focus groups; vignettes describing an adolescent with depression, behavioural problems, and psychotic symptoms; address suggested advice helpful services (b, c) semi-structured interviews; address experiences with services, expectations, suggested improvements	15 (medium)
Brown (2014) [73]	20	3–4	USA	Black (75%) Mothers (90%) Urban	Primary care centre	Parental concerns about emotion/behaviour problems	Prior referral to behavioural health services (70%); followed through with referral (45%)	Interviews; questions related to experiences of discussing problem behaviour with primary care doctor, thoughts and feelings related to referral and decision to follow through or not with referral	18 (high)
Bussing (2012) [43]	161	14–19	USA	African American (28%); white (72%)	Longitudinal study; school screening	High risk ADHD (75%; elevated ADHD ratings/previous diagnosis) low risk ADHD (25%)	previous ADHD treatment (58%)	Open ended question on written survey; undesirable aspects of treatment	16 (high)
Chapman (2014) [56]	16	12–18	USA	Latino (100%)	Part larger state-wide study immigrant youth and their parents			Interviews; scenarios covering range symptoms (depression, PTSD, behavioural disorders); questions relating to views about these behaviour difficulties and help seeking	15 (medium)
Cohen (2012) [61]	24	<19	USA	Mothers (92%) Spanish speakers (67%)	Part of evaluation of child health insurance program		Previous mental health service use	Focus groups and interviews; questions relating to decision to seek care, response from professionals, impressions, and experiences of services	10 (low)
Crawford and Simonoff (2003) [65]	30	6–17	UK	Mothers (70%) White (70%) Black (23%) Asian (7%) Urban	School for emotionally and behaviourally disturbed Random subsample from larger study	Emotional/behavioural disorder problems (and special educational needs)		Focus groups; questions relating to previous experience with a range of services (including child and adolescent mental health services), barriers to accessing services and areas for improvement	18 (high)
dosReis (2010) [42]	48	6–18	USA	Mothers (75%) Urban	Primary care clinics; developmental and behavioural pediatric clinics; specialty mental health outpatient clinic	ADHD diagnosis	Within one month of diagnosis; on medication or still deciding treatment	Semi-structured interview; address understanding of child’s problems and diagnosis, perceptions and expectations of treatment	18 (high)
Flink (2013) [69]	41	10–20	The Nether-lands	Mothers (100%) Dutch (26.8%), Moroccan (31.7%) Turkish (41.5%)	Migrant organisations, mosques and schools		7% reported mental health care past year	Focus groups;questions based on vignette describing internalising problems in adolescent girl and stages of help seeking (including barriers and facilitators)	15 (medium)
Gerdes (2014) [75]	73	5–12	USA	Latino (100%) Urban	Parishioners at local Catholic churches			Written response to open ended questions included in the ‘Problem Recognition Questionnaire for ADHD’, including possible barriers to seeking help, and ways to overcome barriers	17 (high)
Goncalves (2012) [54]	6	12–17	Portugal	None born in Portugal	Schools in areas with high number of immigrants; immigrant organisations			Two focus groups; address access to mental health care for migrant and ethnic minority families, including concepts of mental health, barriers/facilitators to help seeking and service access	12 (medium)
Guzder (2013) [68]	20	7–12	Canada	Immigrant (50%); native born (50%)	Psychiatric hospital patients	Externalising disorders (CBCL)	Psychiatric day hospital (100%)	Semi-structured interview; address help-seeking process, and experiences with services or support	10 (low)
Klasen and Goodman (2000) [66]	29		UK		Specialist services; support groups; community services	Hyperactivity diagnosis/waiting to see specialist	Accessed specialist service/waiting to access	Semi-structured interviews; views of hyperactivity, perception of GP views, treatment options, sources of information	18 (high)
Lindsey (2013) [58]	11		USA	African American (100%) Urban	Two elementary/middle schools			Focus groups; address help seeking, school and community mental health services, barriers and facilitators to help seeking	17 (high)
Meredith (2009) [49]	16	13–17	USA	Hispanic/black/White	Purposive subsample from larger sample recruited through primary care waiting room, followed by diagnostic assessment	Depression diagnosis	Previous treatment (approximately 50%)	Semi-structured interview; include questions about barriers to care	10 (low)
Messent and Murrell (2003) [78]	7		UK	Mothers (57%) Bangladeshi (100%) Urban	Child and adolescent mental health service users with positive view of service		Current or recent mental health service users	Two meetings; views about low rate of referral to specialist mental health services among Bangladeshi families	7 (low)
Murry (2011) [46b]	21	13+	USA	Mothers (100%) African American (100%) Rural	Subsample from follow-up in longitudinal study	Borderline/clinical range on CBCL subscale	25% reported seeing someone for child’s emotional/behaviour problem	Semi-structured interview; address identification of child problems, beliefs about illness, formal and informal support systems, experiences with behavioural health systems, and community resources	18 (high)
Pailler (2009) [55]	59	12–18	USA	Mothers (65%) African American (70%)	Emergency department in hospital			Semi-structured interview; address views relating to depression screening in emergency departments, acceptability of depression screening, and barriers/facilitators to following through with referral	18 (high)
Pullman (2010) [56]	8	4–17	USA	Mothers (75%) White (88%) Rural	Referred to service for severe emotional problems	Severe emotional problems	Referred to specialist service (100%); attended service (50%)	Semi-structured interview; experience of referral and service, and associated benefits and challenges	18 (high)
Sayal (2010) [45]	34	2–15	UK	Black or ethnic minority (52%) Urban	Community based organisations; schools; GPs parents with concerns about child’s mental health	SDQ abnormal range (86%)	Previous mental health service user (9%)	Focus groups; address barriers and facilitators to accessing care	18 (high)
Semanksy (2004) [67]	68		USA		Two states with comprehensive state child and adolescent mental health services	Serious emotional disturbance		Focus groups; address experiences of seeking treatment	12 (medium)
Stein (2003) [71]	UK	Mothers (100%) Pakistani (50%) White (50%) Urban	Urban health centre 79 participants complete questionnaire, subsample respond to open-ended questions					Open-ended questions as part of questionnaire related to help-seeking intentions, advantages and disadvantages of attending services, improvements to services	8 (low)
Thompson (2013) [74]	32	13–19	USA	Mothers (100%) African American Urban	Purposive subsample from larger longitudinal study of mother–child dyads from area of high poverty/high rates child protection service use		Reported mental health service use (100%)	Semi-structured interview; address experiences, expectations and intentions to use mental health services	18 (high)

*n* number of parents/caregivers, *age range* age range of children/adolescents, *CBCL* Child Behaviour Checklist, *SDQ* Strengths and Difficulties Questionnaire

The studies varied widely on a number of characteristics, including country (with the largest number from the USA); age range (with variable age range, and some focusing on younger/older age groups); demographic profile (with some urban or rural populations, and some studies of immigrant groups or particular ethnic/racial groups); method of recruitment and study settings (with samples recruited through various community settings or through mental health service providers); mental health status of participants (with samples of parents of children with mental health problems/diagnosis or without mental health problems); nature of mental health problem (with some studies focused on mental health problems, in general, and others focused on specific mental health problems); and extent of mental health service use (with samples of current/previous service users or referrals, those with a history of help-seeking/prior receipt of a mental health diagnosis, non-service users, a minority of service users/varying levels of service use, or service use was not reported).

Studies providing quantitative data tended to measure parental perceived barriers using a questionnaire that asked participants to either endorse the presence or absence of barriers from a list or rate barriers on a 3–5 point Likert response scale. Some quantitative studies, however, asked more open questions about the reasons for not seeking help or difficulties associated with seeking help/attending services/accessing services. Only two quantitative studies provided data relating to perceived facilitators of accessing mental health services [37, 38]. The amount of relevant quantitative data reported across studies ranged from data relating to responses to a single question [39, 40] or particular questionnaire subscales [23], through studies reporting a breakdown of responses to a large number of questionnaire items [26, 38, 41].

Qualitative data relating to perceived barriers and facilitators tended to be collected using interviews and/or focus groups, with a minority using written questionnaires. All qualitative studies provided data relating to perceived barriers, and 13 provided data relating to perceived facilitators. Like quantitative studies, the amount of relevant data provided by qualitative studies varied considerably, with perceived barriers/facilitators to treatment access only forming a very small part of some studies [42, 43], and the primary focus of others [44, 45].

### Quality ratings

As shown in Tables 1 and 2, quality ratings of quantitative studies ranged widely from 8 to 19 (out of a possible 20); and corresponding ratings of qualitative studies similarly ranged from 7 to 18 (out of a possible 18). Research questions, study design, participant selection, and sample size were mostly assessed positively for quantitative studies; whereas methods of data collection, analyses, and reporting of findings specifically in relation to perceived barriers/facilitators were areas of weakness across lower quality studies. Evidence of robust development and evaluation of the measure of barriers/facilitators among the target population was lacking across all quantitative studies.

Similarly, research questions, study context, and overall study design were mostly well described and appropriate across qualitative studies, but the barrier/facilitator data collection methods and data analysis were often not clearly described among lower quality studies, and the credibility of the findings among these studies was often limited.

### Quantitative and qualitative data synthesis

**Fig. 2:**
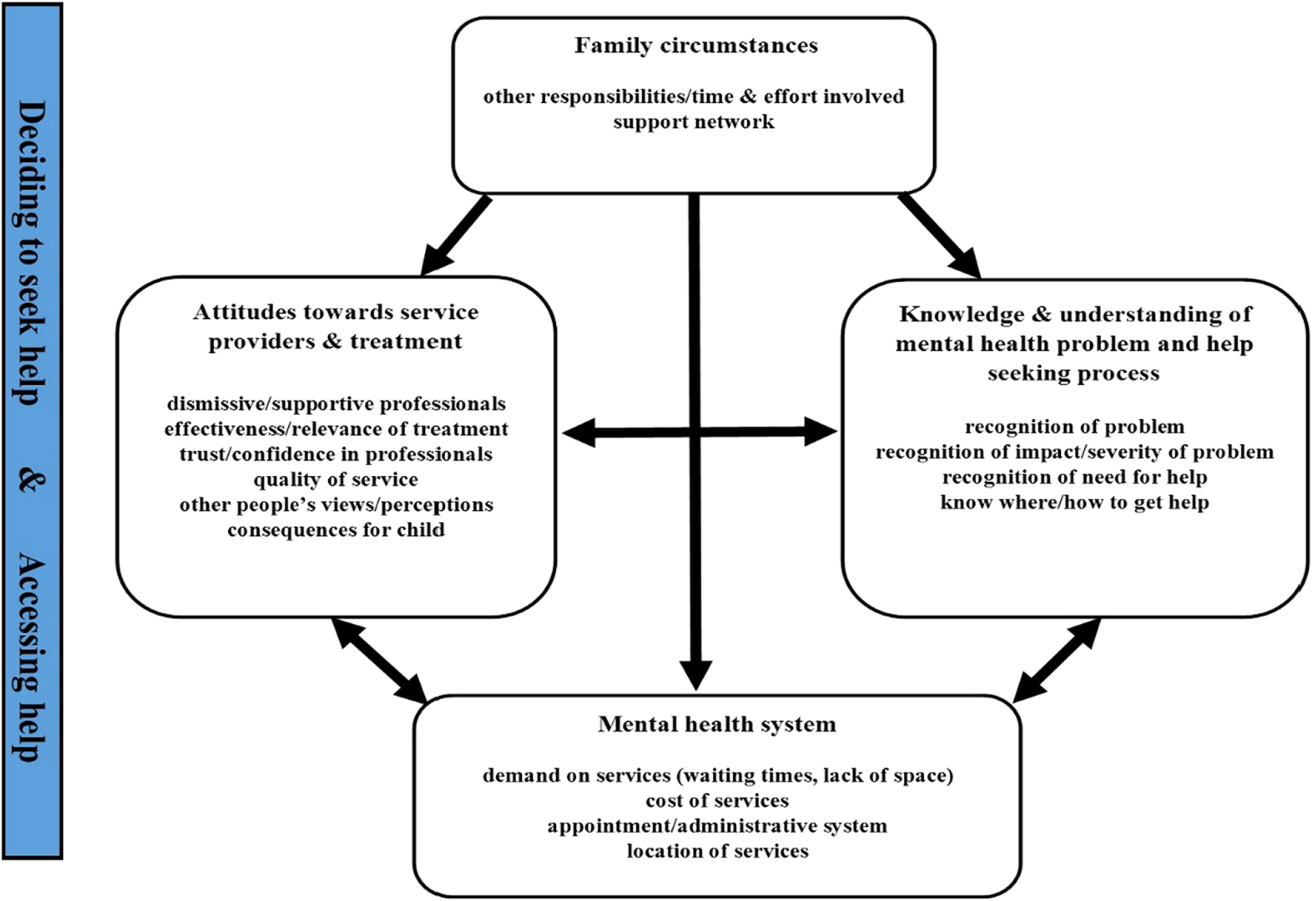
Perceived barrier/facilitator themes

As illustrated in Fig. 2, perceived barriers and facilitators relating to four inter-related themes emerged: (1) systemic and structural issues associated with the mental health system; (2) views and attitudes towards services and treatment; (3) knowledge and understanding of mental health problems and the help-seeking process; and (4) family circumstances. Perceived barriers/facilitators within each theme are summarised below^1^ and outlined in detail in Electronic supplementary material 4.

### Systemic-structural barriers and facilitators

**Fig. 3:**
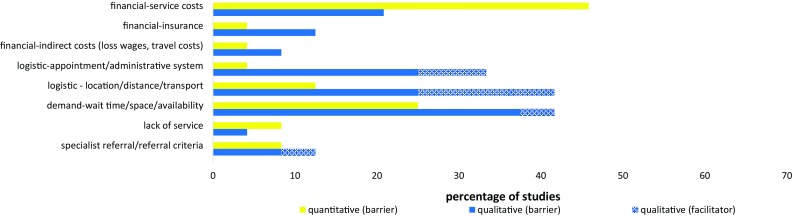
Perceived systemic-structural barriers and facilitators: Percentage of quantitative* and qualitative** studies to report each barrier/facilitator. *Percentage of quantitative studies = Percentage of 24 included samples where a ‘medium’ (10-30) or ‘large’ (>30) percentage of participants endorsed the barrier/facilitator. **Percentage of qualitative studies = Percentage of 24 included studies that reported the barrier/facilitator

Figure 3 illustrates the range of barriers and facilitators relating to systemic-structural aspects of mental health services that were reported across quantitative and qualitative studies.

The cost of mental health services was reported to be a barrier by more than 10% of participants across almost half of quantitative studies [26, 37, 46–53]; and among a smaller number of qualitative studies [54–58]. With a few exceptions, these studies were all conducted in USA and participants were typically not mental health service users. Other financial barriers identified in fewer quantitative and qualitative studies included a lack of insurance coverage (in USA studies) and indirect costs (e.g., loss of wages and travel costs).

Various logistical-type barriers and facilitators were identified. Quantitative studies often asked participants to rate ‘inconvenient (appointment) times’ as a possible barrier, although typically, only a small minority of participants rated this as a barrier [38, 41, 53, 59]. Qualitative studies also identified the cumbersome administrative system [56] and various aspects of the appointment system [44, 45, 57, 61] as perceived barriers/facilitators. Both quantitative and qualitative studies highlighted the location of service providers and the availability of transport as logistical barriers for some families; and the potential benefit of providing logistical support for families was also noted in qualitative studies.

The demands on services, and in particular, the wait to access services were a recurring systemic-structural barrier reported across quantitative [41, 49, 51, 52, 64] and qualitative [44, 55, 60, 61, 65–69] studies from different countries, particularly among samples of service users. Studies also identified a complete lack of specialist services and referral criteria as perceived barriers/facilitators.

### Attitudes towards service providers and psychological treatment

**Fig. 4:**
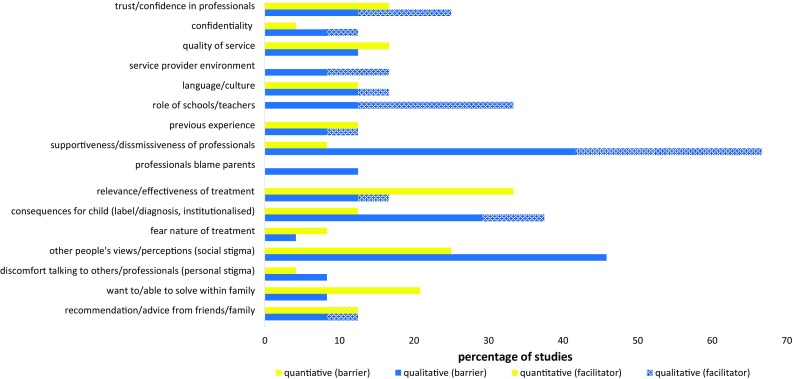
Perceived barriers and facilitators related to attitudes towards service providers and psychological treatment: Percentage of quantitative* and qualitative** studies to report each barrier/facilitator. *Percentage of quantitative studies = Percentage of 24 included samples where a ‘medium’ (10-30) or ‘large’ (>30) percentage of participants endorsed the barrier/facilitator. **Percentage of qualitative studies = Percentage of 24 included studies that reported the barrier/facilitator

Figure 4 illustrates the wide range of views and attitudes relating to professionals, different elements of service providers, and the consequences of seeking and receiving psychological treatment that were identified as barriers/facilitators across studies.

Trust and confidence in professionals and the existence/absence of a trusting relationship with professionals were reported as a barrier/facilitator in both quantitative [26, 38, 46, 70] and qualitative studies [45, 55, 60, 63, 66, 71]. Concerns surrounding confidentiality of discussions with professionals, broader perceptions of the nature, and quality of services, and the previous experience with services were also identified as perceived barriers/facilitators among quantitative and qualitative studies. A perceived language or cultural barrier/facilitator was specifically reported among samples of minority populations; and the service provider environment and specific views towards teachers/schools emerged as potential barriers/facilitators in qualitative studies.

The attitudinal barrier reported by parents in the largest number of (predominantly qualitative) studies was the feeling of not being listened to or dismissed by professionals. A sense of parents feeling dismissed emerged among 10 (42%) qualitative studies [42, 45, 46, 60, 61, 66, 67, 69, 73, 75]; and several qualitative studies [45, 61, 66] also reported that parents felt ‘blamed’ by professionals. On the other hand, a quarter of qualitative studies [45, 46, 55, 58, 61, 75] reported that perceiving that health professionals listen to voiced concerns encouraged parental help-seeking.

Various beliefs surrounding the consequences of help-seeking, for example, the relevance/effectiveness of treatment, the potential consequences for the child, and fears associated with the treatment itself were all identified among some studies as posing barriers/facilitators to help seeking. The most commonly reported barrier related to concerns surrounding the consequences of help seeking, however, was the barrier posed by the perceived negative attitudes among other people. The ‘stigma’ associated with mental health problems or attending mental health services was reported as a barrier in studies from different countries and cultures, including 11 (46%) qualitative studies [45, 54, 55, 57, 58, 60, 61, 63, 69, 71, 75], and among at least 10% of participants in six (25%) quantitative studies [40, 41, 46, 47, 49]. More ‘personal stigma’ or negative self-evaluation among parents, and discomfort talking about a child’s difficulties; a desire to solve problems within the family; and the role of advice from family/friends, were also all highlighted as deterring or encouraging help seeking in several quantitative and qualitative studies.

### Knowledge and understanding of mental health problems and the help-seeking process

**Fig. 5:**
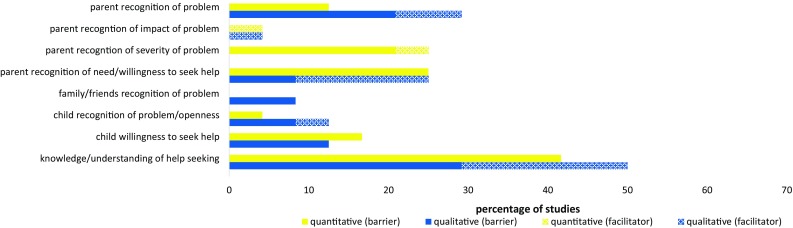
Perceived barriers and facilitators related to knowledge and understanding of a child’s mental health problem and the help-seeking process: Percentage of quantitative* and qualitative** studies to report each barrier/facilitator. *Percentage of quantitative studies = Percentage of 24 included samples where a ‘medium’ (10–30) or ‘large’ (>30) percentage of participants endorsed the barrier/facilitator. **Percentage of qualitative studies = Percentage of 24 included studies that reported the barrier/facilitator

Figure 5 illustrates that the barriers and facilitators reported across studies relating to awareness and understanding of both child mental health problems and the process of seeking professional help for these problems.

Parental recognition of (1) the existence of a child’s mental health problem, (2) the severity of the problem, and (3) the associated impact was each reported as perceived barriers/facilitators to help seeking among a number of studies. Similarly, between 12 and 26% of parents reported not wanting/not needing help across a quarter of quantitative samples [38, 49, 50, 70, 76], and recognition of the need for help or parental willingness to seek help was similarly cited as barriers/facilitators to help seeking in a number of qualitative studies [44, 58, 74, 75, 78]. A lack of family recognition, the presence/absence of recognition by the child themselves, and a child’s own reluctance to seek help were also reported as helping/hindering help seeking in some studies.

Among 10 (42%) quantitative samples [26, 41, 47, 51–53, 59, 70, 77], at least 14% (and up to 75%) of participants reported a lack of knowledge about where or how to get help as a barrier. This lack of knowledge about where to go to ask for help and how to go about getting help was corroborated in a number of qualitative studies [45, 56, 60, 78]. Qualitative studies [45, 46, 55, 58, 61, 63, 69, 71, 73] also highlighted that wider parental understanding of the mental health system also acted as a barrier/facilitator to help seeking.

### Family circumstances

**Fig. 6:**
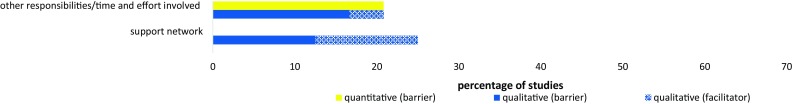
Perceived barriers and facilitators related to a family’s circumstances: Percentage of quantitative* and qualitative** studies to report each barrier/facilitator. *Percentage of quantitative studies = Percentage of 24 included samples where a ‘medium’ (10–30) or ‘large’ (>30) percentage of participants endorsed the barrier/facilitator. **Percentage of qualitative studies = Percentage of 24 included studies that reported the barrier/facilitator

As displayed in Fig. 6, other barriers/facilitators reported in studies related to additional specific aspects of family circumstances, including other responsibilities and commitments, and the time commitment involved in help seeking; and the family’s support network.

### Robustness of data synthesis

Studies assessed to be of low quality (six quantitative studies and five qualitative studies) were removed, and barrier/facilitator codes and themes were re-examined. This sensitivity analysis showed that the overall synthesis remained unchanged when limited to higher quality studies only.

## Discussion

This review synthesised findings from 44 studies addressing parental perceptions of barriers/facilitators to seeking and accessing help for mental health problems in children and adolescents. Perceived barriers/facilitators related to four key themes emerged across studies (displayed in Fig. 2).

In relation to systemic-structural issues surrounding the mental health system, the demand on services emerged as a perceived barrier internationally, reported in studies conducted in the UK, USA, Australia, and Canada. Importantly, waiting times and difficulty getting a referral were most commonly reported as barriers among samples of service users, suggesting that it is after some experience of waiting to access services (or experiencing difficulty accessing services) that these issues often become most pertinent to families. In contrast, the barrier posed by the cost of services (or associated insurance issues) was most frequently reported among community samples in USA, suggesting the ‘threat’ of paying fees to access services can actually deter families from attempting to seek help at all. Other indirect costs associated with service use, such as loss of wages and travel costs, were less commonly reported as barriers within and across studies, but, nevertheless, highlight how certain family circumstances (e.g., living in a rural area) may increase the likelihood that aspects of the mental health system present a barrier to access. Equally, findings indicated that some parents perceive logistical aspects of mental health systems (such as the appointment/administrative system and the location of services) as both barriers and facilitators to seeking and accessing help—but the wide variation in the frequency with which these issues were reported across studies highlights how both variations in mental health systems (e.g., presence/absence of flexible appointment systems/convenient services) and variation in family circumstances (e.g., access to transport and time available to attend appointments) may influence the likelihood that parents perceive such issues as barriers.

A range of views and attitudes towards services and treatment emerged as perceived barriers/facilitators, and notably, these views and attitudes often appeared to be shaped by the previous experience with the mental health system (or contact with services/professionals more generally). In particular, feeling not listened to or dismissed/blamed by professionals was frequently reported as a barrier to seeking and accessing help across qualitative studies; and equally, the perceived benefit of ‘supportive’ professionals was also evident. Similarly, trust and confidence in professionals, views surrounding the quality of services, and views relating to specific professionals (e.g., teachers, GPs) were all identified as presenting barriers/facilitators to both seeking and accessing help across diverse samples. Other attitudinal barriers/facilitators related to the consequences of treatment also emerged, including beliefs surrounding the effectiveness or relevance of treatment, fears surrounding the negative consequences of treatment, and fears associated with treatment itself. However, more notable was the frequency with which parents across studies reported the detrimental impact of perceived negative attitudes of others (as well as personal discomfort surrounding mental health) on help seeking.

Knowledge surrounding both mental health problems and the help seeking process emerged as perceived barriers and facilitators across a wide range studies. The large number of studies—and the large number of participants within some studies—that reported barriers related to not knowing where or how to seek help was particularly salient. Interestingly, among studies that addressed recognition of a child’s mental health problem, relatively large numbers of parents reported perceived difficulties identifying a problem (or a child’s lack of recognition) as a barrier to seeking help, and similarly, parents’ perception of the importance of recognition of the severity and impact of a problem was also clear in some studies.

Perceived barriers/facilitators relating specifically to family circumstances, such as other commitments or responsibilities and a family’s support network, were less commonly directly addressed in studies than other types of barriers/facilitators. Nevertheless, these issues were raised in qualitative studies, and reported by a sizeable minority of participants in several quantitative studies, thus highlighting the role family circumstances can play. Moreover, the potential impact of other aspects of a family’s circumstances (e.g., prior contact with mental health services, living in a rural area, access to transport, language spoken) on the experience of other types of barriers was also clearly illustrated.

### Implications

This review highlights several key areas of potential intervention to minimise barriers to help seeking to improve rates of treatment access for mental health problems in children. In relation to mental health systems, it is evident that ensuring service provision is sufficient, and available free of charge would remove key barriers to seeking and accessing professional help. Minimising the ‘cumbersome’ nature of mental health systems and offering flexible services would also make seeking help easier for many families (e.g., providing drop-in services in local community settings, such as schools and primary care facilities). Moreover, the potential benefit of ensuring professionals working within the mental health system (primary care, schools and specialist services) have the opportunity and skills to develop trusting relationships with families, adopt a supportive approach, and communicate well with other professionals was equally evident.

In addition to improvements to mental health systems, the potential benefit of targeted approaches to improving public knowledge and understanding of childhood mental health difficulties and the help-seeking process was also illustrated. Equipping parents with knowledge and tools to help them identify mental health problems in children, as well as specifically targeting stigmatising attitudes towards parents and the culture of parental ‘blame’ would help to overcome key barriers to help seeking. Moreover, raising awareness and understanding of the professional help that is available and the process involved in seeking help for childhood mental health problems could help provide families with the necessary knowledge about where and how to seek help, as well as foster positive attitudes towards the potential benefits of psychological treatment.

### Strengths and limitations

By focusing on parents’ own perspective surrounding the help-seeking process, this review importantly extends what is known from research specifically addressing the predictors of service use. Notably, the wide range of perceived barriers/facilitators identified here illustrates the plethora of factors at play in determining the likelihood that a family will access services. Findings from quantitative studies shed light on the number of parents who perceive particular barriers at different stages of the help–seeking process; and qualitative studies provided further detail on the specific nature of barriers and corresponding facilitators, as well as identifying additional issues that were not addressed in questionnaire studies. Variation in findings across studies helped illustrate who may and may not experience particular barriers/facilitators and the relationship between barriers/facilitators across the key themes.

Studies included in the review varied widely in terms of design and primary purpose, the amount of data relevant to the review, participant populations, and measures of barriers/facilitators. While similarities and differences across study characteristics were explored, due to the wide variability in sample characteristics, it was not possible to carry out more detailed sub-group analyses examining factors associated with perceived barriers/facilitators, e.g., the age of the child/adolescent, study setting, child/adolescent mental health status, or the type of mental health problem. Although removing the poorest quality studies from the analysis did not impact on the overall findings, it is also important to acknowledge the wide variation in quality of studies included in the synthesis. The lack of well-evaluated measures of perceived parental barriers/facilitators specifically in relation to help seeking for childhood mental health problems presented a limitation across quantitative studies. Indeed, the fact that barriers/facilitators were reported in qualitative studies that were not addressed in the questionnaires illustrates limitations with existing questionnaire measures. Moreover, a large number of both qualitative and quantitative studies focused on parents of children who had accessed services, and therefore, the review was limited in the extent that it was able to address barriers among families who have not reached services. It is also important to note that the systematic search used to identify studies for inclusion in this review was conducted in October 2014, and therefore, any relevant studies published since this data were not included in the review.

The available literature highlights the need for improvements to child mental health services and interventions to raise public awareness and understanding of childhood mental health difficulties and how to access available services. However, further investigation into parents’ perceptions of barriers and facilitators to seeking and accessing treatment for mental health problems in children and adolescents is needed. Specifically, findings from qualitative studies should inform the development of questionnaire measures to ensure all relevant barriers/facilitators which are captured and can be quantified. For example, qualitative studies have highlighted the need to address parents’ perceptions of the dismissiveness/supportiveness of professionals in barrier/facilitator measures—an area frequently neglected in quantitative studies to date. Studies also need to focus on community populations to develop a fuller understanding of varying factors that help and hinder parents at all stages of the help-seeking process. Closer examination of variation in the perceived barriers/facilitators among parents of children of different ages and across different mental health disorders is also necessary to inform more tailored approaches to improve access to treatment.

## Electronic supplementary material

Below is the link to the electronic supplementary material.

Supplementary material 1 (DOCX 40 kb)

## Footnotes

1 Two quantitative studies reported data relating to perceived barriers/facilitators for two sub-samples (a sample of service users and non-service users [41]; and a sample with depression and without depression [49])—and these sub-samples were treated separately in the following analyses
