# Supplementary material for: What do parents perceive are the barriers and facilitators to accessing psychological treatment for mental health problems in children and adolescents? A systematic review of qualitative and quantitative studies
Source: Eur Child Adolesc Psychiatry. 2017 Jan 4;26(6):623–47. doi: 10.1007/s00787-016-0930-6 (PMC5446558; doi:10.1007/s00787-016-0930-6)
Supplement: Supplementary file 1 — Supplementary material 1 (DOCX 40 kb) [file 787_2016_930_MOESM1_ESM.docx]

Supplementary Material

What do parents perceive are the barriers and facilitators to accessing psychological treatment for mental health problems in children and adolescents? A systematic review of qualitative and quantitative studies

European Child & Adolescent Psychiatry

Tessa Reardon,^1^ Kate Harvey,^1*^ Magdalena Baranowska,^1^ Doireann O'Brien,^1^ Lydia Smith,^1^ Cathy Creswell ^1^

^1^ School of Psychology and Clinical Language Sciences, University of Reading, UK

*Correspondence to Dr Kate Harvey (k.n.harvey@reading.ac.uk)

Supplementary Material 1

*Search PsychInfo, Embase & Medline using NHS Evidence Healthcare Database*

Search: Title / Abstract

Limits: English, Article

Search terms: Group 1 AND Group 2 AND Group 3 AND Group 4

| Group 1 | Group 2 | Group 3 | Group 4 |
| --- | --- | --- | --- |
| Barrier*  Hurdle  Obstruct*  Obstacle  Promot*  Facilitat*  Encourage*  Support*  Cause*  Predict*  ‘Unmet need’  Hinder  Willingness  Enable* | Help-seeking  help seeking  helpseek*  ‘Seek* help’  ‘Care seeking’  ‘seek* care’  ‘access treatment’  ‘access service’  ‘seek* treatment’  ‘Service* use’  ‘Service* utilisation’  ‘Service* utilization’  ‘treatment participation’  ‘treatment engagement’ | Mental*  disorder*  Psychopathology  ‘Emotional problems’  ‘Emotional distress’  ‘Behavio* problems’  Internali*  Anxi*  Affect*  Depress*  Suicid*  Externali*  ADHD  ‘self harm’  Psychosis  Oppositional  Conduct | Child*  Adolescen*  Youth  Parent*  Caregiver*  Carer*  Teen*  Mother*  Father*  Maternal  Paternal |

Supplementary Material 2

*Inclusion and exclusion criteria*

Inclusion criteria:

1. Study reported parents’/ caregivers’ perceived barriers or facilitators to accessing psychological treatment for mental health problems in children or adolescents
2. Study addressed mental health problems; emotional and/or behavioural problems; or a specific mental health disorder
3. Study participants were parents/caregivers (i.e. primary carers) of children/adolescents

Exclusion criteria:

1. Study did not report barriers or facilitators to accessing treatment for mental health problems in children/adolescents

2. Study was a review.

3. Study was not published in English in a peer-reviewed journal.

4. Study only reported child/adolescent perceived barriers or facilitators to accessing treatment for mental health problems

5. Study only reported factors associated with or predictors of parent or child/adolescent help seeking behaviour / service use / help seeking intentions

6. Study only reported findings from an intervention designed to address one or more barrier to help-seeking

7. Study only reported perceived barriers/facilitators to accessing ongoing to treatment, not initial access to treatment

8. Study only reported perceived barriers/facilitators to accessing treatment for autism spectrum disorder or developmental disabilities

9. Participants in the study were not parents/caregivers of children or adolescents.

(excluded if the mean age of the children/adolescents was >18 years or if the sample included adults >21 years)

10. Study did not report any qualitative or quantitative data that could be extracted.

11. Study only reported perceived barriers/facilitators to accessing medication or inpatient psychiatric care

12. Participants in the study represented a special population (ie, children/adolescents with an intellectual or developmental disability, young offenders, children/adolescents with mental health problems in the context of a specific physical health condition, looked after children)

13. Study only reported perceived barriers/facilitators to accessing parenting support not specifically targeted at mental health problems in children

Supplementary Material 3

*Quality Rating Checklists*

1. Quantitative studies quality rating checklist

|  | Yes = 2 | Partial = 1 | No = 2 |
| --- | --- | --- | --- |
| Question / objective sufficiently described |  |  |  |
| Study design evident and appropriate |  |  |  |
| Method of participant selection described and  Appropriate |  |  |  |
| Sample size appropriate |  |  |  |
| Participant characteristics sufficiently described |  |  |  |
| Measure of barriers/facilitators well defined |  |  |  |
| Measure robust |  |  |  |
| Analyses described/justified and appropriate |  |  |  |
| Results reported in sufficient detail |  |  |  |

*^Modified version of Kmet et al’s Standard Quality Assessment Criteria [33]^*

2. Qualitative studies quality rating checklist

|  | Yes = 2 | Partial = 1 | No = 2 |
| --- | --- | --- | --- |
| Question/objective sufficiently described |  |  |  |
| Are the research question/s suited to qualitative inquiry |  |  |  |
| Study design well described and appropriate |  |  |  |
| Context of study clear |  |  |  |
| Sampling strategy described, appropriate and justified |  |  |  |
| Data collection methods systematic, clearly described and appropriate for research question |  |  |  |
| Data analysis systematic, clearly described and appropriate for research question |  |  |  |
| Use of verification procedures to establish credibility |  |  |  |
| Are the claims/conclusion credible? |  |  |  |

*^Modified version of Kmet et al’s Standard Quality Assessment Criteria [33], incorporating Dixon-Woods’^*^[34]^ *^prompts^*

Supplementary Material 4

Quantitative and qualitative data synthesis: Detail relating to the perceived barriers/facilitators within each theme

*Systemic-structural barriers and facilitators*

Figure 3 illustrates the range of barriers and facilitators relating to systemic-structural aspects of mental health services that were reported across quantitative and qualitative studies.

*Financial factors*

Questions relating to financial barriers were frequently incorporated into questionnaires, and the cost of mental health services was viewed to be a barrier by more than 10% of participants across almost half of samples (and more than 25% of participants in a quarter of samples). All but two of these studies were conducted in the USA [26, 37, 46, 47, 48, 49, 50, 51], with the remaining two from Australia [52] and New Zealand [53]. With the exception of one sample of mental health service users [51], participants who frequently rated the cost of services as a barrier were recruited in the community, and were typically not mental health service users. The cost of services was reported as a perceived barrier in a smaller number of qualitative studies, but similarly, with the exception of a study of immigrants in Portugal [54], other qualitative studies were from the USA [55-58] and were mostly with those who had not accessed services.

In addition to the direct cost of services, other financial barriers identified in a small number of quantitative and qualitative studies included a lack of insurance coverage and indirect costs (e.g. loss of wages, travel costs). All four studies that specifically identified insurance issues as barriers were USA studies [37, 49, 55, 56]. Loss of wages was only rated as a barrier by a small number of participants in several samples [38, 41, 59], and was not rated as a barrier by more than 10% in any sample [41]. Participants in both qualitative studies that referred specifically to costs associated with travel, noted the barrier in relation to living in a rural area [56, 60].

*Logistical factors*

As well as financial barriers, various logistical-type barriers and facilitators were identified in studies. Quantitative studies often asked participants to rate ‘inconvenient (appointment) times’ as a possible barrier, however, with the exception of one sample [51], only a small minority of participants in other samples (less than 8%) [38, 41, 53, 59], rated this as a barrier. Qualitative studies, on the other hand, pointed towards several wider – but related - issues that presented hurdles to treatment access. As well as appointment times (e.g. ‘their hours aren’t very flexible’ [57], pp. 8), qualitative studies also identified the cumbersome administrative system [56], the need to make multiple telephone calls to access specialist services [61], and the system for making appointments and length of appointments with primary care doctors as barriers [45]. Equally, authors of qualitative studies also noted that parents identified ‘flexible services’ [44], being able to make appointments easily and being given time to discuss concerns about their child’s mental health [45] as potential facilitators.

Both quantitative and qualitative studies also highlighted the location of service providers and the availability of transport as logistical-barriers for some families. The number of participants in quantitative studies who rated lack of transport and the location of services as a barrier varied greatly from more than 40% of participants [62] through to only 1% [59]. Both distance to services, and lack of transport were also identified in qualitative studies as barriers for some parents; and were not restricted to studies of rural populations [49, 55]. The potential benefit of providing logistical support for families to help them access services was also noted by participants in qualitative studies, (e.g. ‘…arrange transportation for them…Help them get where they need to go…’ [55] pp. 725). In one qualitative study one participant also noted the potential benefit to anonymity of using distant service providers: ‘the location provides us with some confidentiality’ [63] (pp. 2422).

*Demands on services, availability of services and referral criteria*

Another recurring systemic-structural barrier reported across quantitative and qualitative studies related to demands on services, and in particular, the wait to access services. Although the number of participants who rated waiting times as a barrier varied across quantitative studies, almost half of a sample of UK service-users [41], more than a third of a USA sample of parents of children with a ADHD diagnosis [64]; and at least 10% of participants in four other samples considered waiting times to be a barrier [49, 51, 52]. Waiting times were similarly cited as a barrier in qualitative studies across different countries, including the UK [65, 66], the USA [61, 67], and Canada [60, 68]. Notably, waiting for appointments most frequently emerged as a barrier among samples of service users, and the quantitative studies in which only a small number of participants endorsed waiting times as a barrier, involved samples of non-service users (or predominantly non-service users) [26, 41, 59]. Several qualitative studies highlighted that parents perceived the limited number of specialists as the cause for long waiting times [60, 66, 67]. Indeed, one study noted that parents perceived GPs and social workers to be relatively ‘available’ in comparison with psychologists or psychiatrists (‘I always say even if you don't know anything you will always be able to find a GP’ [69] pp. 296). However, as well as limited service provision, a complete lack of specialist services was also perceived as a barrier by participants in one qualitative study [68] and a few quantitative studies [40, 41] – again, including more than 40% of a sample of UK service users [41].

Related to the high demand on services, a few quantitative and qualitative studies also specifically identified referral criteria and difficulties families face being referred to specialist services as perceived barriers. Again, difficulty with referral was most frequently endorsed as a barrier by a UK sample of service users [41]. The rigid intake criteria and the required severity of the problem were cited in qualitative studies as reasons for making it difficult to access help from specialists [44, 60]. Equally, one qualitative study highlighted that the referral criteria only posed a barrier in some situations, and in ‘crisis’ situations ‘jumping the queue’ occurs – and so if a child’s problem meets service criteria, this will facilitate access [60].

*Attitudes towards service providers and psychological treatment*

Figure 4 illustrates the wide range of views and attitudes relating to professionals, different elements of service providers, and the consequences of seeking and receiving psychological treatment that were identified as barriers/facilitators across studies.

*Trust and confidence in professionals*

Trust and confidence in professionals, and the existence – or absence of – a trusting relationship with professionals was reported as a barrier/facilitator in both quantitative and qualitative studies. Among the four (17%) samples asked to rate ‘a lack of trust’ or ‘a lack of confidence’ in professionals, at least 10% of participants endorsed the item as a barrier [26, 38, 46, 70]; and importantly it was given both as a reason for not seeking help [46, 70], and as a reason for not following through with a referral [38]. Similar views were echoed in several qualitative studies [63, 66, 71]; but while Klasen et al [66] cited that ‘parents felt GPs were often badly informed’; a more specific belief that services and professionals would be ‘discriminatory’ was reported in studies of ethnic minority populations in the UK [63, 71]. On the other hand, Pailler et al [55] reported that a belief in professionals’ ‘expertise’ was perceived as a facilitator among some parents. Similarly, two other qualitative studies highlighted the potential benefits of having a good and trusting relationship with health professionals. Boydell et al [60] cited familiarity and the ‘long-established relationships’ with service providers in rural communities as facilitators; while Sayal et al [45] specifically highlighted the positive influence a good relationship with a primary care doctor can have on help seeking: ‘I’m lucky in that I’ve got a very good family doctor . . . I would feel personally fairly happy to go with my son to him because I’ve got the trust in my GP – maybe I’m just fortunate perhaps’ (pp. 481).

*Confidentiality*

A concern surrounding the confidentiality of discussions with professionals was also identified as a barrier by participants in a small number of quantitative and qualitative studies. However, while 15% of a sample of service users reported worries about confidentiality as a barrier [41]; fewer participants in (predominantly) non-service user samples endorsed the same barrier [41, 59]. Nevertheless, similar concerns that professionals ‘cannot keep a secret’ [63] (pp. 2417) and the fact that in rural communities ‘everybody knows when mental health service is sought for a child’[60] (pp. 184) were cited as deterrents to seeking help in two qualitative studies; interestingly, professionals maintaining confidentiality was also reported by parents in one study as important for adolescents themselves as well as parents [69].

*Quality of services and service provider environment*

In addition to views focusing on professionals, and relationships with professionals, quantitative and qualitative studies also identified the role of broader perceptions surrounding the nature and the quality of services available. The absence of good quality of care was endorsed as a barrier among 12-19% of participants in several samples [41, 49], and more specifically, poor communication between professionals providing services [41] and a lack of communication between service providers and families [72] were endorsed as barriers by almost half of two samples. However, quality of care and communication between service providers were far less frequently reported as barriers in two other samples of non-service users [41, 59]. Qualitative studies identified as barriers both a general dissatisfaction with services (e.g. ‘didn't give us anything that was terribly helpful’ [44] pp.137), and frustration with being passed from one service to another [45]. The service provider environment was also reported in qualitative studies to either encourage or discourage families from attending services, with the ‘clinic environment’ cited as a barrier by participants in one study [71], and the need to attend specialist services outside a primary care setting cited as a barrier in another study (‘I don’t trust just leaping into another physician or leaping into another office that we have no history with’ [73] pp. 417). Similarly, a ‘comfortable’ physical environment and seeking help in a ‘welcoming environment’ were identified as facilitators in two qualitative studies [46, 71].

*Language/cultural factors*

Another negative perception of service providers reported as a barrier among a few samples was a perceived language or cultural barrier. More than a quarter of a sample of Chinese parents living in the UK cited a language barrier as deterrent to help seeking [39], and 13% of a predominantly Hispanic sample in the USA [64]. Similarly, over 30% of Murry et al’s [46] sample rated white professionals as not understanding the problems of African Americans. Qualitative studies of minority populations reported parallel findings, with Spanish speakers in the USA citing language as a barrier both in relation to communicating with professionals and to understanding the mental health system [61]; and immigrants in Portugal [54] and Pakistani parents in the UK [71] also referred to language difficulties as a barrier. Additionally, Stein et al [71] noted that parents also referred to ‘language facility’ and an awareness of ‘religious knowledge and appreciation’ among staff as a potential facilitators.

*Role of schools/teachers*

As well as views relating to health care professionals and services, specific views and attitudes towards teachers and the role of schools also emerged from qualitative studies as posing potential barriers and facilitators in the help seeking process. A perception that teachers lack expertise and training in mental health was reported as a barrier in three (13%) qualitative studies [58, 61, 68], but on the other hand, the role teachers can play in identifying difficulties [46, 58, 69] providing mental health information [61] and helping to facilitate subsequent help seeking [54] were also reported.

*Previous experience*

The role prior experience with service providers can play in shaping these pertinent attitudes and views was evident across findings that highlighted the importance of these attitudes. Several quantitative and qualitative studies also specifically identified ‘previous experience’ as either a barrier or facilitator to future help seeking. Negative experiences associated with previous attempts to seek help or previous service use were rated as a barriers by 11-18% of participants in three samples [41, 53, 59], and fewer than 10% in a further two samples [26, 41]. Similarly, previous negative experience was identified as a reason for giving up seeking help [45] and shaping future expectations and help seeking behaviour [58]; but equally a positive help seeking experience among another family member was cited as a reason for subsequent help seeking for a child in another qualitative study [74].

*Supportiveness/dismissiveness of professionals*

The attitudinal barrier reported by parents in the largest number of studies was the feeling of not being listened to or dismissed by professionals. Interestingly, only a relatively small number of quantitative studies asked participants to rate not being listen to by professionals as a barrier, however, when posed with this exact question, more than half of a sample of UK service users rated it as a barrier [41], whereas very few non-service users in the same study rated it in this way [41]. Similarly, Hickson et al [70] reported that 10% and 16% of participants respectively reported doctor ‘unwillingness’ and appearing ‘too busy’ as reasons for not sharing a psychosocial concern with a paediatrician. However, a stronger sense of parents feeling dismissed emerged among 10 (42%) qualitative studies [42, 45, 46, 60, 61, 66, 67, 69, 73, 75] (e.g. ‘I’m trying to tell them now that children don’t normally bite themselves and pull their own hair out. My daughter’s doing this and no one will listen’ [67] pp. 24; ‘You see no one understands, no one believes you. It's like smashing your head against a brick wall’ [66] pp. 200). Boydell et al [60] reported parents feeling their expertise as caregivers was ‘ignored and undervalued’, and Sayal et al [45] similarly reported that parents felt GPs and health visitors did not take them seriously (e.g. ‘I’ve spoken to him on about five occasions. It’s always been brushed under the carpet.’[45] pp. 480). Studies also referred to parents’ frustration when doctors attributed their child’s behaviour to a ‘phase’ [46] or suggested behaviours were normal and age-appropriate behaviours [73]. However, in contrast, a quarter of qualitative studies [45, 46, 55, 58, 61, 75] reported that perceiving that health professionals are understanding and empathetic, and that they listen to voiced concerns encouraged parental help seeking. For example, a parent in one study reported a positive experience when ‘They [mental health professional] are interested in what’s going on, you know, and ask questions of the child or the parent. And, suggesting things and wait for a response from either one of them’ [58] (pp. 115). Cohen et al [61] also emphasised the importance of primary care physicians ‘validating’ parent concerns, and Sayal et al [45] similarly described that parents reported cases where GPs sympathised and gave families time as positively impacting help seeking.

*Professionals blaming parents*

Three (13%) qualitative studies also reported that parents felt ‘blamed’ by professionals, particularly when parents shared concerns about their child, professionals focused on their parenting or the parent’s own mental health. For example, Klasen et al [66] reported that parents described situations where GPs attempted to reassure them or give parenting advice, and that they felt criticised by this and were discouraged from seeking help in the future. Similarly, a parent in Sayal et al’s [45] study reported being described as an ‘over-anxious parent’ by her GP, and this caused her to avoid returning to the GP again.

*Relevance/effectiveness of treatment*

As well as perceptions and views surrounding available services and associated professionals, beliefs related to the consequences of seeking and receiving psychological treatment were also identified across many studies as posing barriers and facilitators to help seeking. Parental beliefs surrounding the relevance and usefulness of treatment were addressed in a number of quantitative studies, including a perception that no-one could help with their child’s difficulty, an assumption that their child’s problem would improve without treatment and that treatment would not be beneficial. Almost 80% of participants who identified at least one barrier to help seeking in Pavuluri’s et al [53] study, endorsed the barrier that the ‘problem would get better by itself’, and approximately one third of the same sample reported ‘no-one could help’ and ‘seeking help was not appropriate because of the child’s age’ as barriers. Almost half of Sayal et al’s [41] sample of service users rated the ‘perception no-one could help with their child’s problem’ as a barrier, although fewer (16%) of the non-service users reported the same barrier [41]. Similarly, concerns that treatment may not help and doubts about the usefulness of treatment were reported as barriers by 10-25% in three further studies [40, 59, 62]. A related belief that a child’s problem would get better without treatment was rated as a barrier to seeking help by 36% of a community sample with a child with ADHD [47], and was rated as a barrier to following through with a referral by 30% in school-based suicide screening study [76]. Other quantitative studies also reported that beliefs that treatment was not relevant or could not help were rated as barriers, but only by a small number of participants [23, 26, 38, 50, 51, 77]. The role of views surrounding the relevance and usefulness of treatment was also highlighted in the findings of several qualitative studies. Stein et al [71] reported that some parents believed treatment would not help or could be detrimental, and another study reported parents’ specific concerns surrounding possible negative consequences of behavioural therapy, including creating conflict between a parent and child [43]. On the other hand, Lindsey et al’s [58] findings highlighted that positive expectations surrounding treatment encouraged parents to seek help (e.g. ‘if it’s gonna be positive, if it’s gonna work, I wouldn’t have a problem with sending my child to [a therapist] to get the help’ [58] pp. 112).

*Negative consequences for a child*

Related to views surrounding the effectiveness of treatment, was the belief that receiving treatment may have negative consequences for a child – and in several quantitative studies such a belief was reported as a deterrent to seeking help. In particular, concern that a child would be labelled and it would be left on their record was rated as a barrier by more than 20% of parents in both a USA and a UK sample of service users [41, 51], but by fewer participants in two non-service user samples [37, 41]. However, with the exception of one USA study where 34% of the sample identified a fear that the child would be taken away as barrier [48], fewer than 5% rated the same concern as a barrier in other quantitative studies [41, 53, 59]. Qualitative studies provided further evidence that fears surrounding possible stigmatisation for a child deters some parents from seeking help, with repeated reference to possible impact on a child’s future, (e.g. ‘many application forms ask if your child has ever received counseling’ [43] pp. 98; ‘You’re labelled so therefore you stunt your own life, you can't get some jobs...’ [74] pp. 8) Sayal et al [45] also highlighted that fear of a diagnosis was also reported by parents to stop them from seeking help, and equally a fear their child would be taken away was a barrier for others, while concern a child would be ‘institutionalised’ was reported in another sample [73]. Nevertheless, in two qualitative studies [45, 66], the potential positive impact of receiving a diagnosis was also identified, both in relation to helping a child to receive appropriate outside support and in motivating a parent to follow expert recommendations.

*Fears surrounding the nature of treatment*

As well as perceived negative consequences of receiving treatment, fears surrounding the treatment itself were also reported as barriers in a few studies. More than one third of those participants who reported a barrier to help seeking in Pavuluri et al’s [53] sample endorsed ‘afraid what treatment would be offered’ as a barrier, and another quantitative study reported that 13% of participants endorsed a fear that seeking treatment would result in a child receiving medication [38]. A similar fear of ‘medicating’ a child was reported to deter some parents from seeking any help in a qualitative study [73].

*Social and personal stigma*

The most commonly reported barrier related to concerns surrounding the consequences of help seeking, was the barrier posed by the perceived negative attitudes among other people. The ‘stigma’ associated with mental health problems and attending mental health services, and worries about what other people would think were reported as barriers by participants in quantitative studies across various countries and cultures, including, among others, half of a USA sample [46a], more than a quarter of a United Arab Emirates sample [40], and 22% of a UK sample [41]. Nevertheless, it is notable that in a quarter of quantitative samples, concern about what others would think and other people’s attitude were rated as barriers by less than 7% of participants [26, 38, 41, 51, 53, 59]. Findings from qualitative studies elaborate on some of the specific concerns parents have about the perceptions of friends/family or others in the community and the impact of ‘courtesy stigma’ or stigma by association. Such concerns include a fear of being judged as a bad parent (e.g. ‘that they would say the problem starts with the parents’[75] pp. 508), fear of gossip (‘The fear of what people will say….if you tell one friend, she will just tell a couple more’ [63] pp. 2417) and feeling embarrassed or ashamed [45]. Indeed, as well as reported ‘social stigma’ or ‘courtesy stigma’ (i.e. concern about other people’s views of parents of children with mental health problems) several studies also identified more ‘personal stigma’ or negative self-evaluation as a barrier, and parents’ discomfort surrounding talking to others about their child’s mental health problems. Among quantitative studies that addressed parental discomfort surrounding discussing a child’s mental health problems, it tended to be only a small minority of participants who endorsed this as a barrier [37, 53, 70, 77], though 12% of participants who reported a barrier in Pavuluri et al’s [53] study rated ‘hating answering personal questions’ as a barrier. Two qualitative studies also referred specifically to the view that talking to others about mental health problems was ‘culturally inappropriate’ and therefore prevented parents from seeking help [45, 71].

*Solving problems within the family and advice from family/friends*

Closely related to reports of social and personal stigma, were references to a desire to solve problems within the family, rather than seek outside help. Wanting to solve the problem alone or within the family was rated as a barrier by 17-42% across three quantitative studies [26, 48, 76]; and similarly feeling able/strong enough to manage the problem alone was rated as a barrier by more than half of those who reported barriers in Pavuluri et al’s [53] study and by 20% in Sawyer et al’s [52] study. Qualitative studies of ethnic minority groups in the UK again reported a similar view that mental health problems should be ‘kept in the family’ [63, 71]. Linked to both a reported desire to solve problems within the family, and concerns surrounding other people’s views, was the reported role of recommendations and advice from family and friends – to either seek help, or to not seek help. Two (8%) quantitative studies reported that family not recommending or supporting help seeking was a barrier for 10-15% of participants [26, 38]; but conversely over half of participants in Harwood et al’s [37] study reported ‘someone telling them they needed help’ as a facilitator. Similarly, both Lindsey et al [58] and Brown et al [73] reported that family members criticising parents for asking for help and recommending that they do not seek help presented barriers for some parents, but equally family members giving parents a ‘push’ to seek help was reported as a facilitator in another qualitative study [44].

*Knowledge and understanding of mental health problems and the help seeking process*

Figure 5 illustrates the barriers and facilitators reported across studies relating to awareness and understanding of both child mental health problems and the process of seeking professional help for these problems.

*Parent recognition of the problem, and its impact and severity*

Parental understanding (or lack of understanding) of their child’s mental health problem was not typically included in quantitative studies; however, among studies that did include items relating to a lack of parent recognition of a child’s mental health problem, relatively large numbers of participants did endorse this as a barrier. More than half of one sample reported that thinking their child did not have a problem was a barrier [76], 38% in another study endorsed ‘reluctance’ to recognise the problem as a barrier [40], and more than a quarter in another sample reported thinking their child’s behaviour was appropriate for their age deterred help seeking [38]. Parental reluctance to acknowledge or ‘denial’ that a mental health problem exists was similarly reported in several qualitative studies as a perceived barrier [58, 73, 75]; and the ‘invisible’ nature of mental health problems making them difficult to detect was highlighted in another qualitative study [60]. Equally, parental recognition that a child’s behaviour was not ‘normal’ was also reported as important in facilitating help seeking [44], but interestingly parental recognition in terms of a ‘deficit’ rather than ‘madness’ was also reported as important in one qualitative study [63]. As well as recognising that a mental health problem exists, recognising the impact of a mental health problem was also reported to facilitate help seeking in one qualitative [44] and another quantitative study [38]. Boulter and Rickwood [44] reported that recognising the impact on the family was a motivator to seek help, and almost 80% of participants in Larson et al’s [38] sample reported that recognising the interference in school, friendships and family life, and an effect on a child’s future and stress in their own life all facilitated help seeking. Similarly, a view that a child’s problem was not severe enough for treatment was rated as a barrier by participants in a number of quantitative studies, ranging from as many as 52% of participants [76], to less than 10% [72, 77]. Moreover, more than 80% in another sample reported that viewing a problem as ‘out of control’ was a facilitator to help seeking [37].

*Parent recognition of the need for professional help*

Similarly, between 12 and 26% of parents reported not wanting or not needing help as a barrier across a quarter of quantitative samples [38, 49, 50, 70, 76]. Messent and Murrell [78] reported that a small number of parents were reluctant to acknowledge children had difficulties that needed outside help; and parents disagreeing with a referral was cited as a barrier in another qualitative study [75]. Conversely, being ready to receive help [75], being open to the prospect of getting help [74], recognising the importance of getting help [58], and recognising they are not able to manage a problem alone [44, 74], were all cited as facilitating parental help seeking in qualitative studies.

*Family recognition and child recognition/willingness*

In addition to parental recognition, (a lack of) family recognition along with absence of recognition by the child themselves, were also reported as barriers in several studies. Family members denying or ‘normalising’ the mental health problem was reported as a deterrent to help seeking in two qualitative studies [44, 73]. The child’s lack of recognition of their own problem was only addressed in one quantitative study, but reported as barrier by almost 30% of participants [76]. Two qualitative studies highlighted a child’s reluctance to talk openly about their difficulties as hindering parent recognition [58, 69]; but equally a ‘trustful bond’ between an adolescent and mother was reported as encouraging a young person to talk to a parent who could in turn seek help [69]. A child not wanting to attend a mental health clinic was included as possible response in several questionnaire studies, and reported as a barrier by between 10 and 22% of participants [26, 38, 51, 62]. Similarly, a child’s ‘resistance’ to attend services or talk to professionals was reported as a barrier in two qualitative studies [54, 61], and the role of adolescents’ own responsibility in initiating help seeking was reported in another [45].

*Knowledge and understanding of help seeking*

In comparison to knowledge and understanding of the mental health problem itself, barriers related to a lack of knowledge surrounding where and how to seek help were more frequently reported across quantitative and qualitative studies. Among 10 (42%) samples, at least 14% of participants reported a lack of knowledge about where or how to get help as a barrier, and as many as 75% of one sample endorsed this barrier [41], and more than 30% in a further four samples [41, 47, 53, 70]. This lack of knowledge about where to go to ask for help and how to go about getting help was corroborated in a number of qualitative studies [45, 56, 60, 78]. Qualitative studies also highlighted that a lack of understanding surrounding what to expect from services and treatment [58, 73], and a limited awareness of how the ‘mental health system’ works [69]; as well as doubts surrounding whether GPs are the appropriate person to ask for help [45], all acted as barriers to parental help seeking. Moreover, a number of qualitative studies also identified the potential benefit of providing parents with information about where to seek help and the referral process in order to facilitate help seeking [46, 55, 61, 63, 71].

*Family circumstances*

As displayed in Fig. 6, other barriers/facilitators reported in studies related to additional specific aspects of family circumstances, including other responsibilities and commitments, and the family’s support network. A ‘lack of time’ was rated as a barrier by almost a third of the sample in one quantitative study [62], and 18% in another [53]; and similarly other responsibilities and being ‘busy’ were endorsed as barriers by 18-24% in a further three samples [49, 72]; although similar barriers were endorsed by fewer than 8% in another quantitative study [38]. Barriers related to child care were also reported by a small minority in two quantitative studies [37, 38]. Two further quantitative studies reported that a minority of parents reported barriers relating to work commitments, other family responsibilities and stressors, and the time involved in treatment [23, 50]. Other priorities and responsibilities, and the time commitment involved, were similarly cited as barriers to help seeking in several qualitative studies [49, 58, 75] (e.g. ‘Maybe she’s focusing on keeping a roof over her head than she actually is on the mental health of the child’ [58], pp.115). Parents in one qualitative study also reported not getting services because they ‘did not push hard enough’ [60], highlighting the effort involved in accessing help – and similarly being ‘demanding’ was reported to facilitate accessing help [60]. Qualitative studies also pointed towards the role of a family’s extended support network in both helping and hindering help seeking. While Lindsey et al [58] and Flink et al [69] reported that some parents relied on support from other church members and church leaders, in place of seeking professional help, feeling ‘socially isolated’ was cited as a barrier to help seeking in another study [57] and receiving informal social support helped some parents ‘overcome barriers’ [75] and learn about available services by ‘word of mouth’ [60].
